# A telomere-to-telomere genome assembly for Meyerozyma guilliermondii ATCC 6260: closing gaps and resolving a translocation in the reference sequence

**DOI:** 10.1099/acmi.0.001091.v3

**Published:** 2025-12-08

**Authors:** Lois L. Hoyer, Brian A. Freeman, Elizabeth K. Hogan, Alvaro G. Hernandez

**Affiliations:** 1 Department of Pathobiology, College of Veterinary Medicine, University of Illinois Urbana-Champaign, Urbana, IL, USA; 2 Roy J. Carver Biotechnology Center, University of Illinois Urbana-Champaign, Urbana, IL, USA

**Keywords:** genome sequence, *Meyerozyma guilliermondii*, Pacific Biosciences (PacBio) HiFi, telomere-to-telomere, yeast

## Abstract

*Meyerozyma guilliermondii,* a haploid yeast (phylum Ascomycota), is useful in bioprocessing and bioremediation, and is an infrequent pathogen of humans. The availability of highly accurate, long-read DNA sequencing methods provided the opportunity to improve the *M. guilliermondii* genome assembly. Using Pacific Biosciences (PacBio) HiFi technology, we generated a chromosome-level, telomere-to-telomere assembly for the *M. guilliermondii* type strain ATCC 6260. This assembly closed gaps in the current reference sequence and resolved a translocation artefact that affected the size and structure of chromosomes 4 and 5. The improved genome sequence is available publicly and will facilitate future studies of this species.

## Data summary

The *Meyerozyma guilliermondii* ATCC 6260 genome sequencing project was assigned the GenBank accession number PRJNA952656, locus prefix QA089, and BioSample number SAMN34081633. The HiFi reads file was deposited in the GenBank Sequence Read Archive under accession number SRR24086009 (SRX19886860). *M. guilliermondii* ATCC 6260 chromosomes were assigned accession numbers CP128815–CP128822. The genome assembly was named ASM5154625v1 (GCA_051546255.1).

## Introduction

The haploid yeast *Meyerozyma guilliermondii* (formerly *Candida guilliermondii* and *Pichia guilliermondii*) is studied because of its importance in bioprocessing (for riboflavin and xylitol production) and in bioremediation [1]. *M. guilliermondii* is also recognized as an infrequent fungal pathogen of humans and was included among the eight species for which genome assemblies were constructed and compared by Butler *et al*. [2]. The focus of that effort was fungal species in the CTG clade, a group that uses the CUG codon for serine instead of leucine [3]. Butler *et al*. [2] also referred to this group of yeasts as the ‘*Candida* clade’, signalling their phylogenetic proximity to, and inclusion of, *Candida albicans*, the most important yeast pathogen. Using this comparative genomics approach, Butler *et al*. [2] conducted extensive analyses of the biology and pathogenicity of the species studied. The *M. guilliermondii* genome assembly that was described in that study was for the type strain ATCC 6260; the assembly serves as the *M. guilliermondii* GenBank reference sequence (ASM14942v1; Ref Seq).

Our interest in the study of the agglutinin-like sequence (*ALS*) family in yeast species closely related to *C. albicans* led us to analyse available genomic resources in an attempt to construct full-length *ALS* genes [4]. Because there are typically several *ALS* genes in each of these yeast species, the 5′ ends of the *ALS* genes are conserved across loci, the *ALS* genes encode numerous copies of repetitive sequences, and *ALS* alleles are divergent within diploid species, genome assemblies typically break within the *ALS* coding regions. Broken *ALS* genes were found even in haploid yeast species such as *M. guilliermondii*. This problem led us to follow the arc of DNA sequence technological advancements, with the goal of deriving the sequences we needed. The availability of highly accurate, long-read DNA sequences, such as those produced using the Pacific Biosciences (PacBio) HiFi technology [5], delivered the data set quality necessary to support accurate assembly of full-length *ALS* genes [6].

Here, we applied PacBio HiFi technology to DNA sequencing of the *M. guilliermondii* type strain ATCC 6260. The result was a chromosome-level, telomere-to-telomere genome assembly that closed gaps and revealed a translocation artefact in the current Ref Seq. The goal of this manuscript is to announce the availability of the new genome assembly and to compare it with the current Ref Seq. Most importantly, this manuscript describes the data sets underlying the new genome assembly and their value for revealing the accurate sequence for any genomic region of interest.

## Methods

All experiments, including genome sequencing, were completed in our laboratories at the University of Illinois Urbana-Champaign. The *M. guilliermondii* type strain ATCC 6260 was purchased from the American Type Culture Collection (Manassas, VA) and immediately stored at −80 °C in YPD medium with 38% glycerol (YPD medium per litre: 10 g yeast extract, 20 g Bacto peptone and 20 g dextrose, with 20 g Bacto agar for plates). The isolate was streaked onto YPD agar and incubated at 30 °C for 24 h for use in experiments. The stock plate was stored at 4 °C for no more than 1 week when a fresh plate was prepared. Each experiment used cells directly from the −80 °C stock to ensure its authentication as the publicly available ATCC 6260 reference isolate.

A single representative colony was used to inoculate YPD medium to grow cells for karyotyping or genomic DNA extraction. The *M. guilliermondii* karyotype was created using the method described by Hoyer [7]. Genomic DNA extraction used a Zymolyase-based protocol [8]. The resulting DNA was treated with RNase (Gold Biotechnology), then with proteinase K (Gold Biotechnology), then extracted with phenol/chloroform and precipitated with 2 vols of 2-propanol. Genomic DNA was resuspended in Tris-EDTA, pH 8.0, and visualized on an agarose gel to confirm that it was >50 kb.

The genomic DNA was sheared with a Megaruptor 3 (Diagenode) to an average fragment length of 13 kb. Sheared DNA was run on a Blue Pippin system (Sage Science) to select 10–50 kb fragments using a 0.75% agarose cassette and DNA marker S1. A library was constructed from the sheared fragments using the SMRTBell Express Template Prep Kit 3.0 (PacBio). The library was sequenced on a single-molecule real-time (SMRT) cell 8M on a PacBio Sequel IIe system using the circular consensus sequencing (CCS) mode and a 30 h movie time. CCS and demultiplexing analysis used SMRT Link v11.1 (ccs –min-passes 3 –min-rq 0.99). The data set included nearly 7.6 billion bp (498,466 reads, with a mean read length of 15,243 bp).

The programme filtlong v0.2.1 [9] was used to select PacBio reads greater than 15 kb and to retain 50× coverage of the predicted 10.6 Mb genome size (--min_length 15000 --target_bases 530000000). The output file was passed to hifiasm v0.16.1 [10], which was run with default parameters. The assembly was constructed on 6 April 2023 and deposited in GenBank on 26 June 2023. Accession numbers were assigned to the genome sequencing project (PRJNA952656; locus prefix tag QA089), HiFi reads file (SRR24086009), and genome assembly (files CP128815 to CP128822, representing the nuclear genome).

A draft annotation was created for the QA089 assembly to provide a roadmap for comparison with the reference sequence (ASM14942v1). Default parameters of funannotate v.1.8.13 were used to clean, sort and mask the chromosomes, then predict coding sequences [11]. Coding regions were translated with the alternative yeast nuclear code using EMBOSS Transeq [12,13], then analysed using InterProScan v5.56–89.0 [14] and eggNOG-mapper web v2.1.9 [15,16]. Output files were passed to the annotate programme of funannotate. SnapGene (https://www.snapgene.com) was also used to translate QA089 chromosomes and visualize potential ORFs.

Versions 0.19.6 and 0.24.0 of hifiasm were subsequently used to create genome assemblies with the same filtlong output file that was used for the initial assembly. BUSCO v5.5.0 [17] was used to assess the completeness of the nuclear genome assembly. blast was accessed online (https://blast.ncbi.nlm.nih.gov/Blast.cgi) or used on the command line to construct custom databases (blast+ v2.16.0). Genome comparisons were made using MUMmer v.4.0.0.beta2 [18]. Minimap2 v2.21 [19] was used to map HiFi reads to chromosomes or scaffolds. The Minimap2 output was processed by SAMtools v1.12 [20] to create and sort the index files for use by the Integrative Genomics Viewer (IGV v2.16.1; https://igv.org) [21].

## Results and discussion

Assembly of the HiFi reads using hifiasm (v0.16.1) revealed eight chromosome-length contigs, each bounded by telomeric repeats (5′-ACTGGTGT-3′). The sequence of the telomeric repeats matched previous reports for *M. guilliermondii* [2,22]. Table 1 compares the features of the GenBank Ref Seq assembly (ASM14942v1) with the new QA089 file. The QA089 assembly reduced the contig number from 71 to 8 and the number of spanned gaps from 62 to 0. Both assemblies indicated a genome size of ~10.6 Mb. The current work was also an improvement over a draft genome assembly we created previously using Oxford Nanopore Technologies MinION long-read and Illumina short-read sequence data (ASM694215v1) [4]. Full details regarding the comparison between that assembly and Ref Seq were published as Data Sheet 2 in the Supplementary Material appended to the manuscript [4]. Numerous point mutations broke ORFs in ASM694215v1, prompting us to generate HiFi long-read data to create the QA089 assembly featured here.

**Table 1.** Comparison between the *M. guilliermondii* ATCC 6260 GenBank reference sequence (ASM14942v1; Ref Seq) and the PacBio HiFi reads-based assembly QA089

Genome feature	Ref Seq	QA089
Sequence length (bp)	10,609,954	10,658,372
Ungapped length (bp)	10,574,537	10,658,372
No. of contigs	71	8^*^
Contig N₅₀ (bp)	313,225	1,721,903
No. of scaffolds	9^*^	8^*^
Largest scaffold (bp)	2,092,950	2,095,439
Scaffold N₅₀ (bp)	1,701,016	1,721,903
No. of spanned gaps	62	0

*A mitochondrial genome sequence was deposited with Ref Seq (scaffold 9; NW_001809792.1; 23,890 bp), comprising the ninth scaffold. Only the nuclear genome was deposited for QA089, representing eight chromosomes.

Newer versions of hifiasm have been released since the QA089 assembly was created, so the same filtered HiFi reads file was processed using hifiasm v0.19.6 and v0.24.0 to determine if there were any major differences in the results (Table 2). Regardless of the hifiasm version used, each assembly featured eight chromosomes bounded by telomeric repeats. The sequences for chromosomes 1 and 6 were identical among all assemblies. Assemblies created with hifiasm v0.16.1 and v0.19.6 were the same, except for an extra 55 bp at the end of chromosome 8 in v0.19.6. Comparisons between the v0.24.0 assembly and the others showed fewer than 10 bp of difference for chromosomes 2, 5, 7 and 8. Larger differences were noted for chromosome 3 (+1,943 bp) and 4 (–16,534 bp) in the v0.24.0 file. The altered region on chromosome 3 featured 11 tandem copies of a 1,692 bp region (predicting a 563 aa protein containing anion exchange protein conserved domains) in the v0.19.6 assembly and 12 copies in v.0.24.0. The 16,534 bp change on chromosome 4 occurred in the ribosomal DNA (rDNA) region, where v0.19.6 placed five tandem copies and v0.24.0 only had three.

**Table 2.** Comparisons between *M. guilliermondii* ATCC 6260 PacBio HiFi reads-based genome assemblies created with different versions of the hifiasm programme

Chr	v0.16.1	v0.19.6	v0.24.0
1	2,095,439	2,095,439	2,095,439
2	1,969,107	1,969,107	1,969,115
3	1,721,903	1,721,903	1,723,846
4	1,357,054	1,357,054	1,340,520
5	1,158,978	1,158,978	1,158,979
6	1,024,188	1,024,188	1,024,188
7	892,426	892,426	892,433
8	439,277	439,332	439,340

Because it was deposited into GenBank first, and because there were few meaningful differences from assemblies created with the newer software, the QA089 assembly (hifiasm v0.16.1) was used for all subsequent analyses. The percentages of complete and single-copy BUSCOs were 96.0% (fungi_odb10), 94.8% (ascomycota_odb10) and 99.4% (saccharomycetes_odb10).

**Fig. 1.**
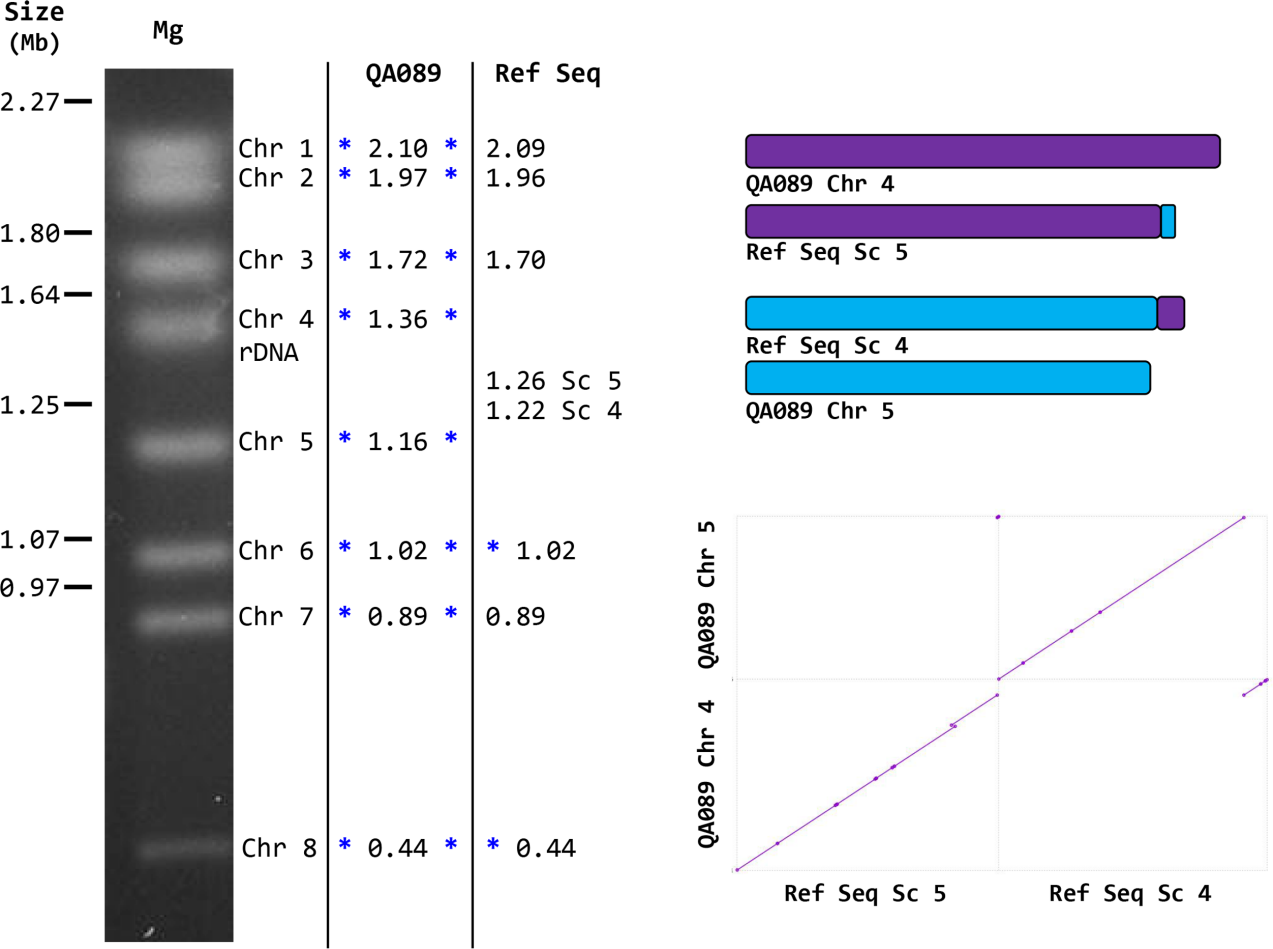
(Left) Ethidium bromide-stained agarose gel showing the electrophoretic karyotype for *M. guilliermondii* ATCC 6260. *C. albicans* SC5314 chromosomes were run on the same gel for use as a size standard (indicated on the left of the image [6]). *M. guilliermondii* (Mg) chromosomes were labelled numerically from the largest to the smallest. Blue asterisks mark chromosome ends where telomere repeats were present. The size of QA089 chromosomes and Ref Seq scaffolds (both in Mb) are indicated on the right of the image, matching them to the karyotype. Ref Seq scaffolds 5 (NW_001809796.1) and 4 (NW_001809797.1) were reversed in order of size, contrary to the largest-to-smallest numbering scheme. These scaffolds corresponded to QA089 chromosomes 4 and 5, as indicated in Table 3. The sizes of the QA089 chromosomes better matched the *M. guilliermondii* karyotype in this region of the gel. (Right) Diagram of the translocation and its effect on scaffold/chromosome size shown as a line diagram (upper) and using a MUMmer dot plot (lower). Translocations occurred near the right end of the Ref Seq scaffolds shown. In the QA089 assembly, sequences with similarity to the Ref Seq gene annotated as *PGUG_03884* were found on both chromosomes 4 and 5. The translocation (assembly artefact) occurred within the middle of *PGUG_03884*.

MUMmer was used to align the Ref Seq and QA089 nuclear genome assemblies (Fig. S1, available in the online Supplementary Material). The Ref Seq scaffolds generally corresponded to *M. guilliermondii* ATCC 6260 chromosomes. Notably, though, Ref Seq scaffold 5 was QA089 chromosome 4, and Ref Seq scaffold 4 was QA089 chromosome 5 (Table 3). QA089 chromosome sizes better matched the electrophoretic karyotype than did the Ref Seq scaffolds (Fig. 1). The MUMmer alignment suggested a translocation in the Ref Seq assembly relative to QA089. Closer examination of this region suggested that the assembly artefact was created in *PGUG_03884* that was annotated in the Ref Seq. Sequences that matched *PGUG_03884* were conserved in similar locations on the QA089 *M. guilliermondii* chromosomes 4 and 5, but were only present on scaffold 4 in the Ref Seq assembly. The Ref Seq assembly was created using a whole-genome shotgun approach that involved end sequencing of plasmid (4 and 10 kb) and fosmid (40 kb) libraries using ABI/Sanger technology [2]. Attempting to assemble this type of data at the ends of two similarly sized chromosomes, each with a region of highly conserved sequence, could easily lead to this type of artefact.

**Table 3.** Comparisons between Ref Seq scaffolds and QA089 chromosomes to show the length differences in the assemblies

Ref Seq scaffolds				QA089 chromosomes			
**Scaffold**	**Contigs**	**GenBank accession**	**Total bp**	**Chr**	**GenBank accession**	**Total bp**	**Δ bp**
1	12	NW_001809800	2,092,950	1	CP128815	2,095,439	+2,489
2	15	NW_001809799	1,964,611	2	CP128816	1,969,107	+4,496
3	12	NW_001809798	1,701,016	3	CP128817	1,721,903	+20,887
5	9	NW_001809796	1,224,156	4	CP128818	1,357,054	n/a*
4	4	NW_001809797	1,255,619	5	CP128819	1,158,978	n/a*
6	8	NW_001809795	1,022,952	6	CP128820	1,024,188	+1,236
7	4	NW_001809794	887,468	7	CP128821	892,426	+4,958
8	6	NW_001809793	437,292	8	CP128822	439,277	+1,985

*Length comparisons were not meaningful because of the translocation between these scaffolds in the Ref Seq assembly and the presence of the rDNA locus on chromosome 4.

na, not applicable.

The accuracy of the QA089 assembly can be assessed by examining the HiFi reads data set: each HiFi read is a piece of raw data that represents contiguous genome sequence from *M. guilliermondii* strain ATCC 6260. The HiFi data set should contain many reads that correspond to QA089 chromosomes 4 and 5, and none that match Ref Seq scaffolds 5 and 4. To explore this idea, *PGUG_03884* was used as the query in a blast search of the HiFi reads file to identify the suspected translocation region on QA089 chromosomes 4 and 5. Ten kb of sequence spanning this site on each chromosome were then used as a blast query for the HiFi reads file. Dozens of HiFi reads were extracted that demonstrated the contiguous sequence of the proposed translocation site on chromosomes 4 and 5 (Supplementary File 1). The same approach was used for the Ref Seq assembly: 10 kb of sequence spanning *PGUG_03884* were used as a blast query for the HiFi reads file. As expected, this region was not represented in a contiguous entirety among the HiFi reads, supporting the conclusion that the Ref Seq is misassembled. Instead, portions of the *PGUG_03884*-containing query matched HiFi reads that mapped to QA089 chromosomes 4 and 5 at the translocation site (Supplementary File 1). These alignments pinpointed the translocation to nucleotide (nt) 1,340 of *PGUG_03884* (which is nt 1,147,225 of the 1,255,619 nt Ref Seq scaffold 4). Fig. 1 includes a cartoon depicting the relationship between these QA089 chromosomes and Ref Seq scaffolds, as well as a dot plot of their alignments.

The gapless, telomere-to-telomere nature of the QA089 assembly provided insight into the contents of the subtelomeric spaces on each *M. guilliermondii* chromosome. There was no more than ~5 kb of sequence missing from the ends of the Ref Seq assembly scaffolds (Fig. S2). In a few instances, these subtelomeric regions contained potential open reading frames (ORFs) that were not annotated previously in the Ref Seq. Examples included oxidoreductase-encoding ORFs on chromosome 1 Left and chromosome 8 Left, an iron transport-encoding ORF on chromosome 2 Right, and an ORF encoding a protein in the cytochrome P450 superfamily on chromosome 6 Left. A family of ORFs predicting Leu-rich proteins was prevalent in the subtelomeric spaces (Fig. S2). Butler *et al*. [2] presented an extensive discussion of *M. guilliermondii* gene families. Gaps that were filled by the new QA089 genome assembly also revealed previously unannotated ORFs in the subtelomeric regions. Examples included ORFs predicting an arginase, a major facilitator superfamily transporter, and 5-oxoprolinase. The new assembly also merged previously distinct ORFs into larger ones. Examples included joining *PGUG_00007* and *PGUG_00008*, as well as joining *PGUG_02286* and *PGUG_02287*. The presence of large ORFs encoding cell-surface adhesins in the *M. guilliermondii* subtelomeric space (e.g. *ALS* and *IFF* genes) was noted previously [4].

The subtelomeric gaps in Ref Seq accounted for much of the size difference between Ref Seq scaffolds and the QA089 chromosomes. The largest size difference was observed for scaffold 3/chromosome 3, which was 21 kb larger in the QA089 assembly (Table 3). Nearly 5 kb of the difference was located at the chromosome ends, while ~15 kb was found 880 kb from the left end. There, the QA089 assembly featured 11 tandem copies of the 1,692 bp ORF (predicting a 563 aa protein with domains conserved with anion exchange proteins) that was mentioned above. The Ref Seq assembly and annotation included four truncated versions of this gene at the same location (*PGUG_02792*, *PGUG_02793*, *PGUG_02794* and *PGUG_02795*), but no full copies.

The quality of the QA089 assembly was also evaluated by examining the coding regions for the *ALS* family that encodes cell-surface adhesion proteins [4]. Sequences for these genes are demanding to assemble because they contain similar 5′ ends and extensive regions of conserved, repeated motifs. Securing complete ORFs for these genes was the main reason for our laboratory group to pursue genome assembly construction. There are four *ALS* genes in *M. guilliermondii: MgALS673* (GenBank MH753516, 2,439 bp) *MgALS2302* (MH753513, 9,681 bp), *MgALS3259* (MH753514, 6,963 bp) and *MgALS3330* (MH753515, 5,469 bp). Only one *ALS* gene was constructed and annotated accurately in the *M. guilliermondii* Ref Seq. Previous efforts involved PCR amplification and Sanger DNA sequencing to construct *M. guilliermondii ALS* coding regions from clues provided by the Illumina MiSeq/Oxford Nanopore MinION-based genome assembly [4]. The new QA089 data provided the opportunity to check these previous efforts against long-read, HiFi data. Each *ALS* coding region matched the previous GenBank deposits except for one nucleotide (nt 218 of *MgALS3259* was A in the Sanger sequence data and C in the HiFi reads data). The HiFi reads-based *ALS* coding regions (with 1 kb of upstream and downstream sequence) were deposited into GenBank (PP209160–PP209163) for easy retrieval.

## Conclusions

PacBio HiFi reads were used to create a chromosome-level, telomere-to-telomere assembly for the *M. guilliermondii* type strain ATCC 6260. The new assembly (QA089) closed Ref Seq gaps and revealed a previously unrecognized chromosomal translocation artefact. Long, difficult-to-assemble genes were intact in the QA089 assembly, providing additional confidence in its accuracy and quality. The QA089 genome assembly improved the available data and data sets for the *M. guilliermondii* type strain, which will facilitate its future study.

## Supplementary material

10.1099/acmi.0.001091.v3 Uncited Supplementary Material 1.

## Abbreviations

*ALS*: agglutinin-like sequence
CCS: circular consensus sequencing
PacBio: Pacific Biosciences
rDNA: ribosomal DNA
SMRT: single-molecule real-time
