# Supplementary material for: A telomere-to-telomere genome assembly for Meyerozyma guilliermondii ATCC 6260: closing gaps and resolving a translocation in the reference sequence
Source: Access Microbiol. 2025 Dec 8;7(12):001091.v3. doi: 10.1099/acmi.0.001091.v3 (PMC12720209; doi:10.1099/acmi.0.001091.v3)
Supplement: Uncited Supplementary Material 1. [file acmi-7-01091-s001.pdf]

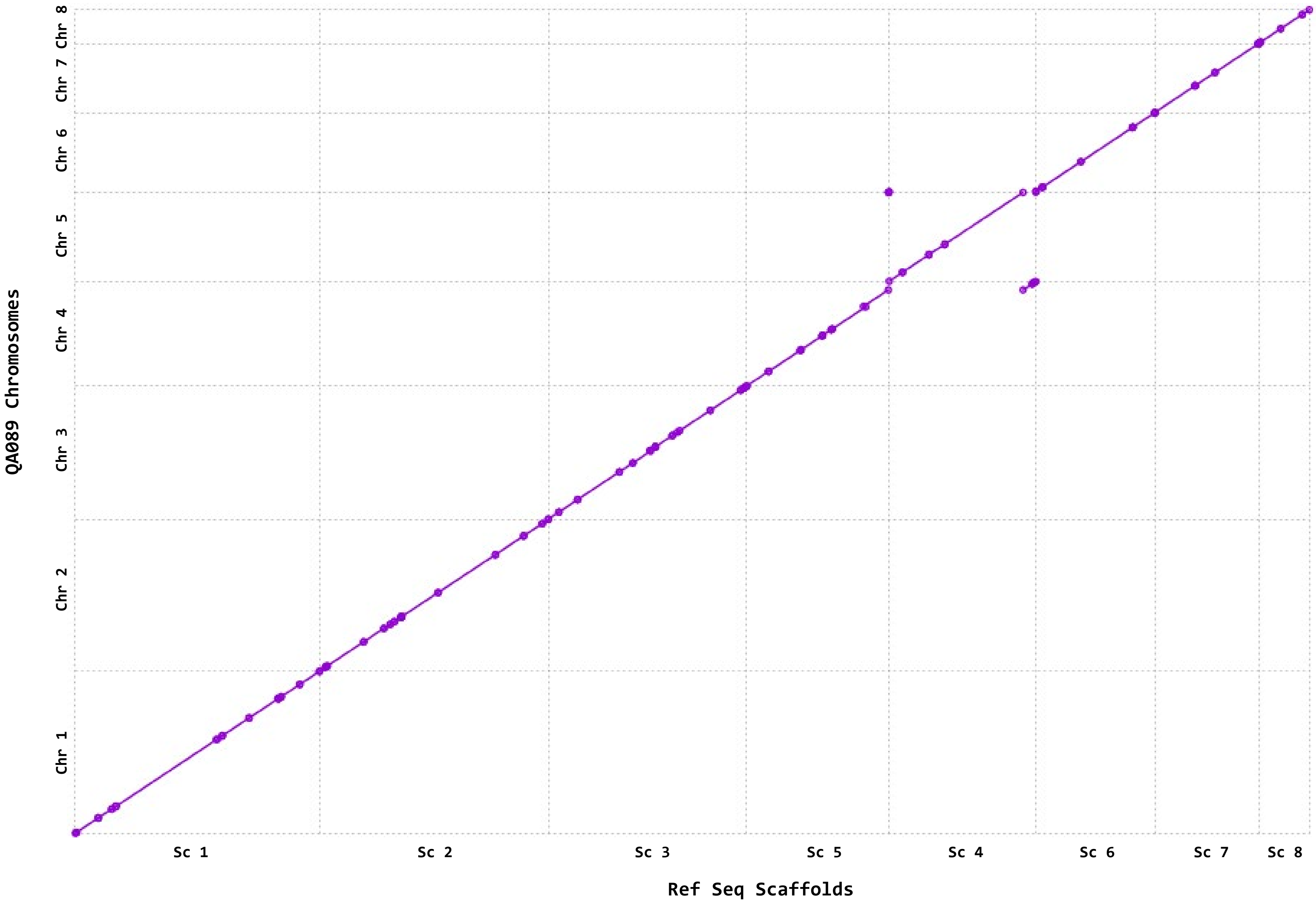

Supplementary Figure 1. MUMmer alignment of the Ref Seq (x axis) and QA089 (y axis) genome assemblies.

Supplementary File 1. Validation of the QA089 genome assembly by identification and mapping of PacBio HiFi reads.

The *PGUG_03884* open reading frame was used to search the QA089 genome assembly to identify regions on chromosomes 4 and 5 where the translocation (assembly artifact) occurred in the Ref Seq. Ten thousand nucleotides from this location were then used as a BLAST query for the PacBio HiFi reads. Identified reads were then mapped to the QA089 and Ref Seq assemblies and visualized using the Integrative Genomics Viewer [21]. Screenshots from the alignments were annotated and presented below.

(A). A BLAST search of the HiFi reads using 10,000 nucleotides from the QA089 chromosome 4 location produced dozens of hits. The top 10 scoring reads and their length (in nucleotides) are listed here. These reads were mapped to the QA089 chromosome 4 and visualized using the Integrative Genomics Viewer. HiFi read identification numbers (e.g. 120195394 as a 19,900-nt read) are shown next to each read in the figure below.

m64108e_230401_152225/120195394/ccs (19,900 nt)
m64108e_230401_152225/80544731/ccs (21,687 nt)
m64108e_230401_152225/11928635/ccs (17,993 nt)
m64108e_230401_152225/69011091/ccs (21,664 nt)
m64108e_230401_152225/22349074/ccs (18,362 nt)
m64108e_230401_152225/14943789/ccs (22,956 nt)
m64108e_230401_152225/14878152/ccs (19,845 nt)
m64108e_230401_152225/43254508/ccs (24,370 nt)
m64108e_230401_152225/148899668/ccs (23,955 nt)
m64108e_230401_152225/110889780/ccs (20,200 nt)

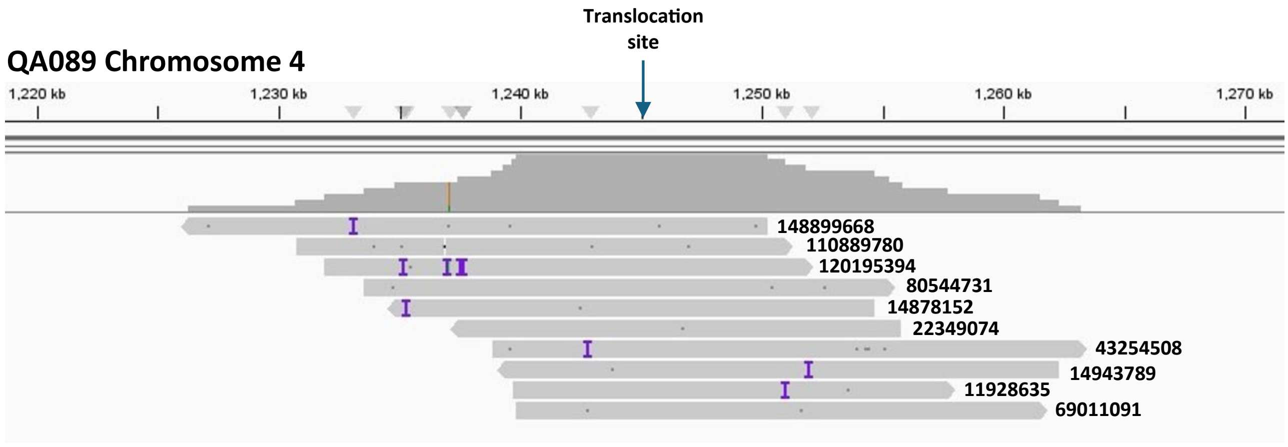

(B). A BLAST search of the HiFi reads using 10,000 nucleotides from the QA089 chromosome 5 location that matched *PGUG_03884* also produced dozens of hits. The top 10 scoring reads are listed here. These reads were mapped to the QA089 chromosome 5. HiFi read identification numbers are shown next to each read in the figure below.

m64108e_230401_152225/173672280/ccs (19,146 nt)
m64108e_230401_152225/167314226/ccs (18,171 nt)
m64108e_230401_152225/131400564/ccs (19,833 nt)
m64108e_230401_152225/91096108/ccs (18,861 nt)
m64108e_230401_152225/159975657/ccs (20,193nt)
m64108e_230401_152225/43124953/ccs (25,010 nt)
m64108e_230401_152225/47254008/ccs (18,527 nt)
m64108e_230401_152225/14223589/ccs (21,323 nt)
m64108e_230401_152225/76743671/ccs(22,651 nt)
m64108e_230401_152225/13240436/ccs (19,735 nt)

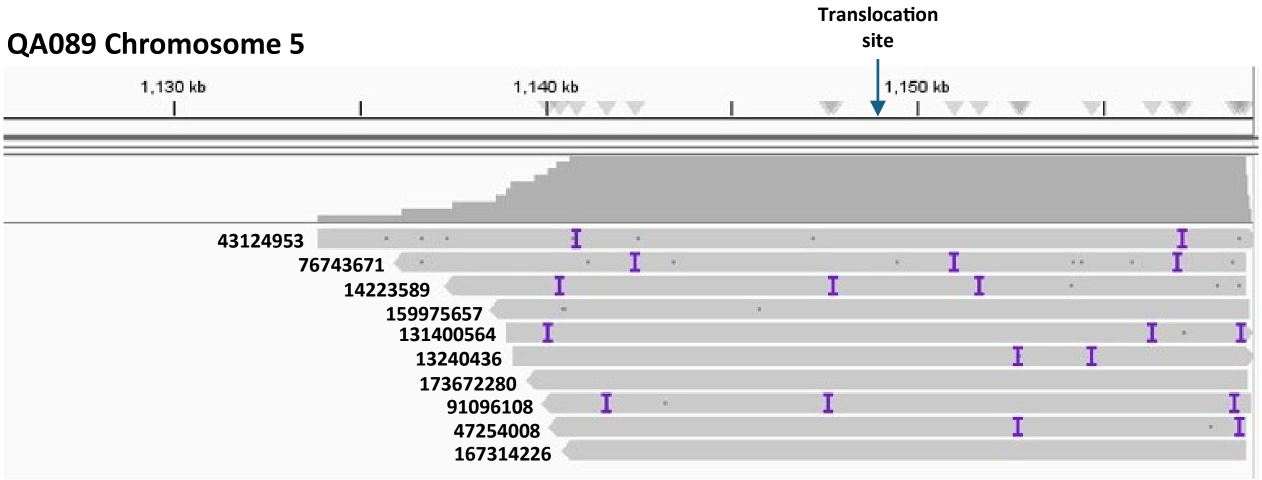

(C). Another BLAST search of the HiFi reads used 10,000 nucleotides that spanned the *PGUG_03884* locus in the Ref Seq assembly. As expected, there was not a single HiFi read that demonstrated these 10,000 nucleotides were contiguous in the QA089 genome assembly. Instead, the search identified dozens of reads that matched nucleotides 1 to 5226 of the search query and dozens of reads that matched nucleotides 5226 to 10,000 of the search query. The identity of these reads matched the ones extracted from the BLAST searches in sections **(A)** and **(B)** above. The 20 reads from **(A)** and **(B)** were mapped to Ref Seq Scaffold 4 to show how they broke at the proposed site of the translocation artifact.

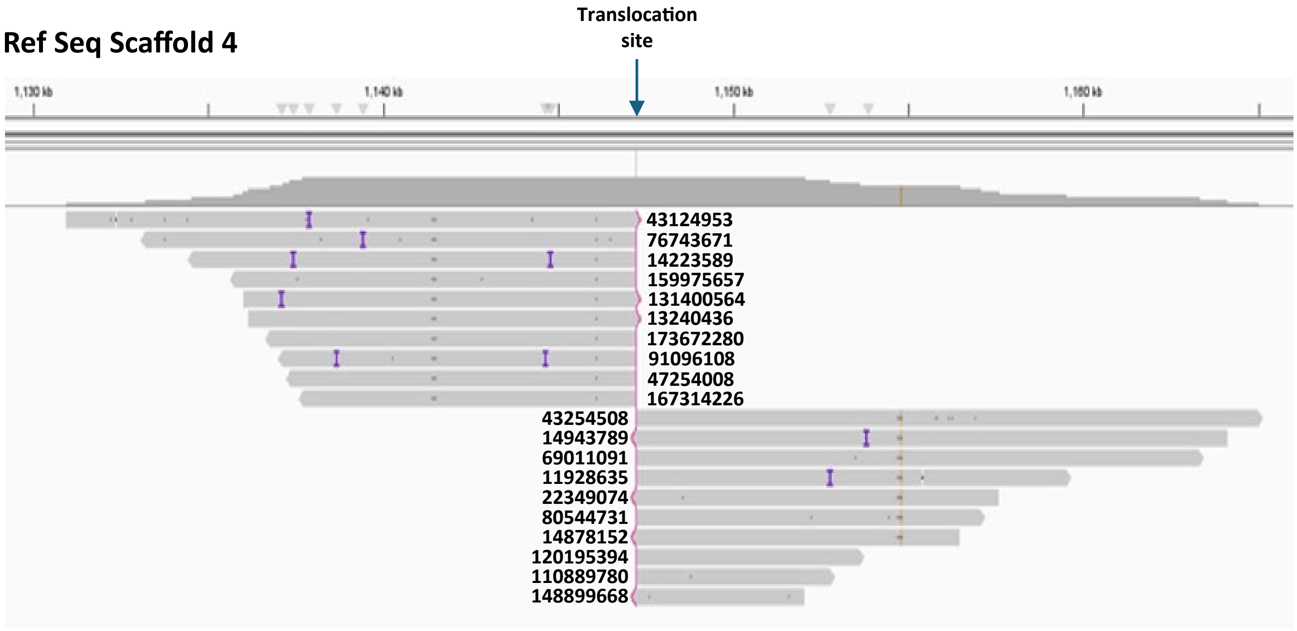

(D). The 20 reads identified in **(A)** and **(B)** were also mapped against Ref Seq scaffold 5. As predicted, they broke at the site of the translocation. The first portion of each read mapped to scaffold 4 and the remainder mapped to scaffold 5.

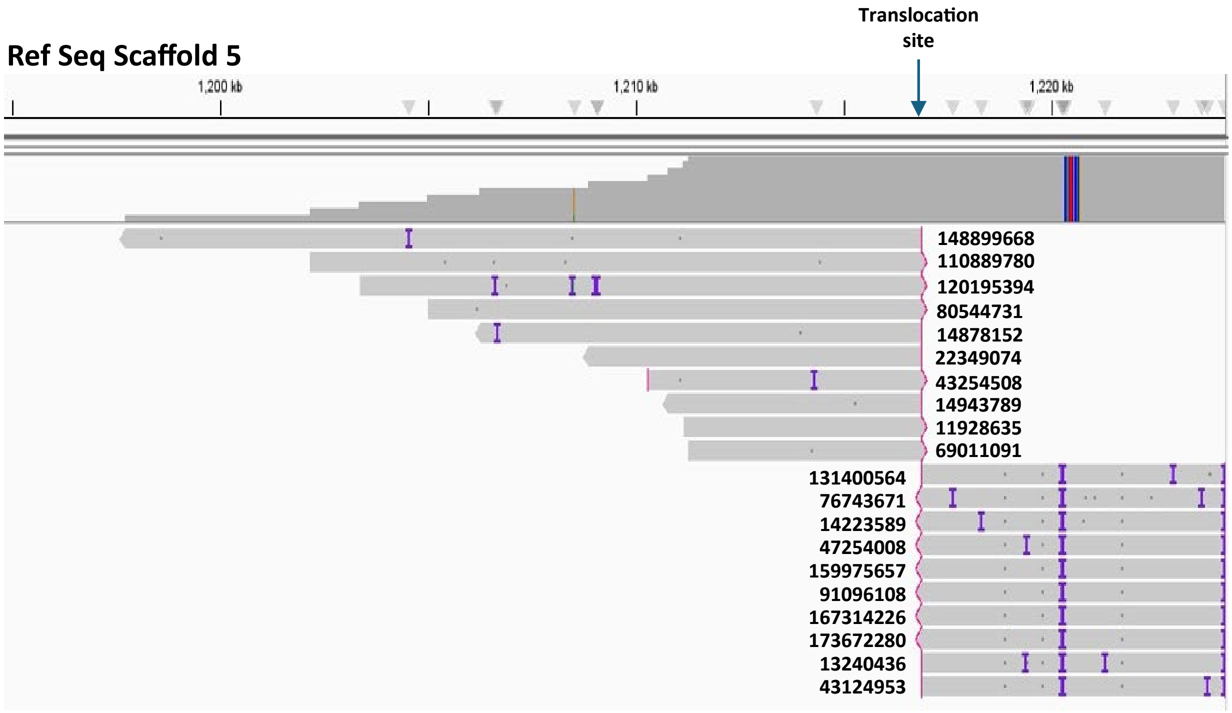

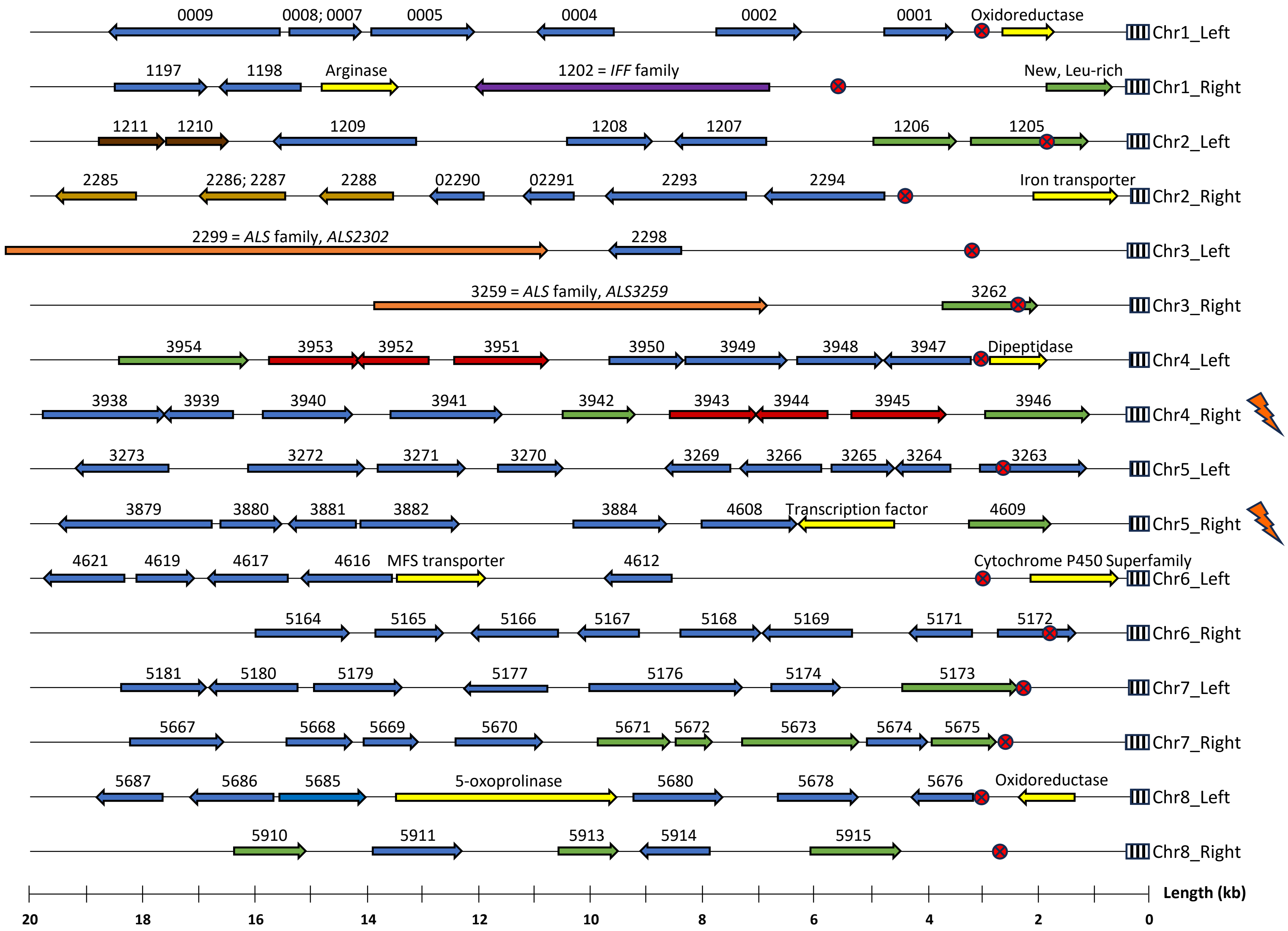

0009
0008; 0007
0005
0004
0002
0001
Oxidoreductase
Chr1_Left
1197
1198
Arginase
1202 = *IFF* family
New, Leu-rich
Chr1_Right
1211
1210
1209
1208
1207
1206
1205
Chr2_Left
2285
2286; 2287
2288
02290
02291
2293
2294
Iron transporter
Chr2_Right
2299 = *ALS* family, *ALS2302*
2298
Chr3_Left
3259 = *ALS* family, *ALS3259*
3262
Chr3_Right
3954
3953
3952
3951
3950
3949
3948
3947
Dipeptidase
Chr4_Left
3938
3939
3940
3941
3942
3943
3944
3945
3946
Chr4_Right
3273
3272
3271
3270
3269
3266
3265
3264
3263
Chr5_Left
3879
3880
3881
3882
3884
4608
Transcription factor
4609
Chr5_Right
4621
4619
4617
4616
MFS transporter
4612
Cytochrome P450 Superfamily
Chr6_Left
5164
5165
5166
5167
5168
5169
5171
5172
Chr6_Right
5181
5180
5179
5177
5176
5174
5173
Chr7_Left
5667
5668
5669
5670
5671
5672
5673
5674
5675
Chr7_Right
5687
5686
5685
5-oxoprolinase
5680
5678
5676
Oxidoreductase
Chr8_Left
5910
5911
5913
5914
5915
Chr8_Right
20
18
16
14
12
10
8
6
4
2
0
Length (kb)

Supplementary Figure 2. Scale diagram of the subtelomeric regions of the *M. guilliermondii* ATCC 6260 chromosomes from the QA089 PacBio HiFi-based genome assembly. The left ends of each chromosome were flipped so that the telomeres (indicated by a striped box) were aligned on the right side of the diagram. The scale bar at the bottom of the figure is calibrated in 1 kb increments. To avoid clutter and to promote an at-a-glance view of the subtelomeric spaces, the diagram focused on open reading frames (ORFs) greater than 1 kb. Arrows show the ORF size and direction of transcription. Translated ORFs were BLASTed against the non-redundant, standard protein database. Numbers above each arrow correspond to the PGUG_0XXXX designation in the current *M. guilliermondii* Ref Seq annotation. Multiple numbers above the same arrow indicate Ref Seq ORFs that were combined into longer ones in the new assembly (e.g. *PGUG_00007* and *PGUG_00008* on Chr 1 Left). Arrow colors other than yellow indicate protein families. Red circles containing an "X" indicate the position where Ref Seq scaffolds ended. There were no more than about 5 kb of sequence missing from the ends of the Ref Seq scaffolds. Lightning bolts at the Right ends of chromosomes 4 and 5 indicate the misassembly of the Ref Seq in these regions relative to QA089.
